# Photonic crystals possessing multiple Weyl points and the experimental observation of robust surface states

**DOI:** 10.1038/ncomms13038

**Published:** 2016-10-05

**Authors:** Wen-Jie Chen, Meng Xiao, C. T. Chan

**Affiliations:** 1 Department of Physics and the Institute for Advanced Study, The Hong Kong University of Science and Technology, Hong Kong, China

## Abstract

Weyl points, as monopoles of Berry curvature in momentum space, have captured much attention recently in various branches of physics. Realizing topological materials that exhibit such nodal points is challenging and indeed, Weyl points have been found experimentally in transition metal arsenide and phosphide and gyroid photonic crystal whose structure is complex. If realizing even the simplest type of single Weyl nodes with a topological charge of 1 is difficult, then making a real crystal carrying higher topological charges may seem more challenging. Here we design, and fabricate using planar fabrication technology, a photonic crystal possessing single Weyl points (including type-II nodes) and multiple Weyl points with topological charges of 2 and 3. We characterize this photonic crystal and find nontrivial 2D bulk band gaps for a fixed *k*_*z*_ and the associated surface modes. The robustness of these surface states against *k*_*z*_-preserving scattering is experimentally observed for the first time.

## Abstract (web summary)

To realize Weyl points carrying topological charges higher than one is challenging. Here, Chen *et al*. report a photonic crystal possessing single and multiple Weyl points with topological charges of two and three using planar fabrication technology.

## Graphical abstract

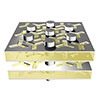

Topological matter such as electronic topological insulators^1^,^2^,^3^,^4^ and their classical wave counterparts^5^,^6^,^7^,^8^,^9^,^10^,^11^,^12^,^13^,^14^,^15^,^16^,^17^,^18^,^19^,^20^,^21^,^22^,^23^,^24^,^25^,^26^,^27^,^28^,^29^,^30^ have attracted a lot of attention. Recently, the attention has shifted towards topological materials that are gapless, exhibiting Weyl nodal points^31^,^32^,^33^,^34^,^35^,^36^,^37^,^38^,^39^,^40^. Weyl points with topological charge of 1 are nodal degeneracy points where two linearly dispersive bands intersect in the three-dimensional (3D) reciprocal space. Weyl point dispersions are governed by the Weyl Hamiltonian 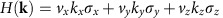, where *v*_*i*_, *k*_*i*_ and *σ*_*i*_ are group velocities, momenta and Pauli matrices. While Weyl points can be viewed as a 3D extension of the two-dimensional (2D) Dirac points 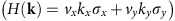, important differences exist. Dirac points can be gapped easily by breaking either their inversion or time-reversal symmetry. However, Weyl points are stable against perturbation since all the degrees of freedom are already exhausted in the Weyl Hamiltonian, so that perturbations respecting the translational symmetry cannot lift the degeneracy but can only shift the position of the nodal points. Their robustness to perturbation stems from the topological invariant (nonzero Chern number) they carry, which can be calculated as either *c*=sgn(*v*_*x*_*v*_*y*_*v*_*z*_) or the integral of Berry curvature (mathematically equivalent to a magnetic field in momentum space) on a closed surface enclosing the Weyl points. These nodal points are quantized sources or sinks of Berry curvature and can be viewed as magnetic monopoles in **k**-space^32^. Weyl points are hence topologically protected and can only be destroyed by annihilation with another Weyl point of opposite topological charge. Their topological character also manifests in the nontrivial surface states connecting the projections of bulk Weyl points in the surface Brillouin zone due to the bulk-surface correspondence.

Weyl points are more elusive entities than Dirac points. The existence of Dirac points is essentially a consequence of the symmetry of the honeycomb lattice and just about any kind of wave, be it electronic^41^, electromagnetic^42^ or acoustic^43^, will exhibit Dirac points at the corner of the Brillouin zone of the honeycomb system as long as inversion and time-reversal symmetries are respective. Simply put, Dirac points are guaranteed if a honeycomb lattice can be made. Such a simple recipe based on symmetry is not available for Weyl points, however. Instead, symmetry reveals the circumstances under which Weyl points cannot exist. For example, time-reversal symmetry requires a Weyl point at k to have the same charge as its companion point at −k, while inversion symmetry requires the pair to have the opposite charge. Such conflicting symmetry requirements preclude the existence of Weyl points in a system with both inversion (*P*) and time-reversal (*T*) symmetries. Such nodal points can in principle exist in various systems with broken *PT* symmetry, but exactly what type of structure can give rise to Weyl points is not known *a priori*. Nevertheless, in the past few years, theory has suggested that Weyl points can be found in a number of systems including cold atoms^44^, layered systems^45^ and also classical wave systems such as photonic^46^,^47^, and acoustic^48^ systems. Many interesting phenomena associated with the existence of the topological point can in principle be found in Weyl systems such as topologically protected surface states^31^, quantum anomalous Hall effect^33^ and chiral anomaly^49^. However, experimentally realizing Weyl points and their associated topological characteristics remains challenging. In particular, the robustness of the topologically protected surface states derived from Weyl points has yet to be observed. So far, Weyl points have been shown experimentally in transition metal arsenide^35^,^36^,^37^ and phosphide^38^,^39^ and a double gyroid photonic crystal^40^. For classical waves, the gyroid structure is very complicated.

In addition, most theoretical discussions of Weyl points focus on single Weyl points carrying a topological charge of 1 as described by the Hamiltonian 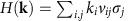. But higher order Weyl points carrying topological charges of >1 can in principle exist in certain types of crystals^50^,^51^. As nodal points, most Weyl points have point-like equienergy or equifrequency surfaces and therefore a zero density of states. However, the so-called type-II Weyl points^52^ can also exist and would have a finite density of states as recently shown theoretically. It is certainly desirable to create a system that exhibits these more exotic variations of Weyl points.

Here we designed and fabricated a photonic crystal that exhibits single, double and triple Weyl points (including type-II Weyl points). The structure was specifically designed to be compatible with planar fabrication technology which, in the microwave regime, can be implemented using printed circuit board (PCB). This is also the first time that robust surface states are measured experimentally at the boundary of Weyl systems.

## Results

### Photonic crystals possessing single and multiple Weyl points

**Figure 1:**
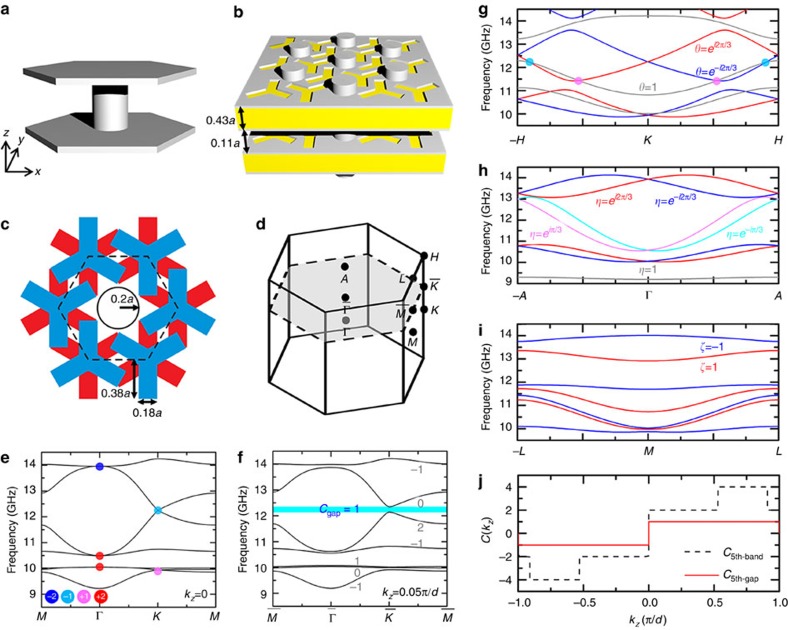
Realization of Weyl points in an electromagnetic system by introducing interlayer coupling. (**a**) Unit cell of a single-layer system built from a hexagonal array of perfect electric conductor (PEC) cylinders bounded by two PEC slabs. This can be realized with metal-coated PCBs that are pierced through by a hexagonal array of aluminium rods. PCBs stacked in the *z* direction form a 3D photonic crystal. (**b**) Multilayer system built from PCBs stacked in the *z* direction. Interlayer couplings are introduced by the Y-shaped slots on two sides of the PCBs. (**c**) Top view of the unit cell (dashed hexagon) of the multilayer system. Blue and red areas highlight the Y-shaped slots on the upper surface and the lower surface of the PCBs. (**d**) Reciprocal space of a hexagonal lattice. Since the photonic crystal has translational symmetry along the *z* direction, *k*_*z*_ is a good quantum number. The system with a fixed *k*_*z*_ has a 2D band structure in the reduced Brillouin zone (grey plane in **d**). Chern numbers are well defined for each *k*_*z*_ slice. Weyl points can be viewed as the phase transition points between the *k*_*z*_ slices with different Chern numbers. (**e**) Bulk band structure in the *k*_*z*_=0 plane. The structure has several Weyl points with different charges (in different colours). (**f**) Bulk band structure in the *k*_*z*_=0.05*π*/*d* plane. (**g**–**i**). Dispersion along the *z* direction at 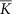, 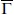 and 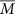, respectively. Bands with different rotational eigenvalues are plotted in different colours. Since a change in rotational eigenvalue results in a change in the band (gap) Chern number, each crossing point in **g** or **h** is a Weyl point whose charge depends on the ratio between the rotational eigenvalues of two intersecting bands. Four Weyl points between the 4th and 5th bands, which induce the jumps in the Chern number of the 5th band in **j**, are highlighted in **g**. (**j**) Chern numbers of the 5th gap (red solid line) and the 5th band (black dashed line) as a function of *k*_*z*_, the jump in which implies the topological charge of associated Weyl points.

Periodic systems exhibiting Weyl points in momentum space can in principle be designed using a nearest-neighbour tight-binding Hamiltonian^48^. Its basic idea can be understood in the following way. It is well known that a honeycomb lattice has 2D Dirac points at Brillouin zone corners *K* and *K*′ and the dispersion can be described by effective Dirac Hamiltonians near these points. To achieve 3D Weyl points, one can stack these honeycomb lattices periodically in the *z*-direction and introduce chiral interlayer coupling (see Methods and Supplementary Fig. 1). Here a tight-binding Hamiltonian is not used as a design tool *per se*, but rather as a starting point to guide us to the structures possessing the correct symmetry to support topological features such as synthetic gauge flux and associated Weyl points. We first design the single-layer system with 2D Dirac points. Figure 1a depicts the unit cell, which when repeated in the *xy* plane will form a hexagonal array of perfect electric conductor (PEC) cylinders embedded in a parallel plate waveguide. The fundamental mode of this waveguide is *E*_*z*_-polarized and it has a conical dispersion at *K* and *K*′. This planar waveguide structure can be realized easily using standard PCB and such PCBs can be stacked in the *z* direction to form a 3D photonic crystal (see Fig. 1b and the detailed geometry in Methods). Then chiral interlayer coupling is introduced by etching slots on the top and bottom PEC layers. Figure 1c shows the top view of the unit cell (dashed hexagon), where the Y slots on the top and bottom surfaces are shown in blue and red, respectively. These slots form a chiral pattern and break all the mirror symmetries and inversion symmetry.

Figure 1d depicts the first Brillouin zone of the photonic crystal, where a grey hexagon highlights the 2D Brillouin zone for a fixed *k*_*z*_. The bulk band structure in the *k*_*z*_=0 plane and along the line from **k**-point −*H* to *H* (passing through the *K* point) are shown in Fig. 1e,g, respectively. The first and second bands, and the fifth and sixth bands intersect at the *K* point, and the dispersions near the degeneracy point are linear in all three directions of the reciprocal space, indicating that they are Weyl points. The form of dispersion (linear or quadratic) around the point of intersection can be obtained by either numerical calculation or symmetry analysis (Methods). The topological charge of a Weyl point can be calculated either by integrating Berry curvature on a closed surface enclosing the Weyl point or by inspecting the rotational eigenvalues of the two touching bands^50^. Here we used both methods to calculate the topological charges and obtained consistent results. The topological charges of the Weyl points at 12.25 and 9.94 GHz at *K* are found to be −1 and 1. The Weyl points at the *K*′ point are found to bear the same topological charges as those at *K*, as mandated by the C_6_ symmetry of the photonic crystal. In addition, we also found three degeneracy points at Γ (shown as red or blue solid circles). Figure 1e,h shows that the band dispersions near these points are linear along the *k*_*z*_ direction but quadratic in the *k*_*x*_–*k*_*y*_ plane. They are double Weyl points^50^ with charges of ±2, which are the superposition of two single Weyl points with the same charges of ±1. Their topological charges can be verified by the number of surface states near these nodal points (see Supplementary Note 1 and Supplementary Fig. 2). To the best of our knowledge, this is the first time that a real structure has been designed and fabricated to exhibit double Weyl points with topological charges higher than 1. Double Weyl points have been predicted to exist in crystals possessing C_4_ or C_6_ point group symmetries^50^. In our case, the degeneracy between the two single Weyl points is protected by C_3_ symmetry and time-reversal symmetry (see the symmetry analysis in Methods).

**Figure 2:**
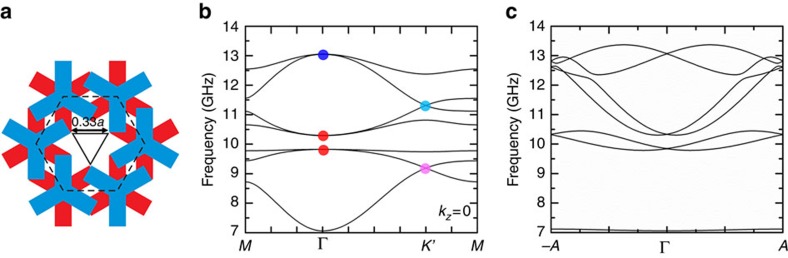
Band structure of a C_3_-symmetric Weyl photonic crystal. (**a**) Unit cell of the crystal where the PEC cylinder is replaced by a triangular rod, reducing the symmetry to C_3_. (**b**) Band structure in the plane of *k*_*z*_=0. All three double Weyl points still lie at Γ. The quadratic dispersions in the *k*_*x*_–*k*_*y*_ plane are also shown near these double Weyl points. (**c**) Band structure from *−A* to *A*, showing the linear dispersion of the double Weyl points in the *z* direction.

To verify that the C_3_ symmetry (together with time-reversal symmetry) is indeed sufficient to protect the double Weyl points, we considered a photonic crystal similar to the one illustrated in Fig. 1c, but with the circular rods replaced by triangular rods. As the triangular rod has a lower symmetry than the circular rod, the replacement reduces the symmetry of the system from C_6_ to C_3_, while maintaining time-reversal symmetry. Figure 2a is the top view of the unit cell, showing that the circular cylinder in the unit cell centre in Fig. 1c is now replaced by a triangular rod, thereby reducing the symmetry to C_3_. Figure 2b plots the band structure in the *k*_*z*_=0 plane. The three Weyl points at Γ have retained their quadratic dispersions. Figure 2c shows the dispersions along the *k*_*z*_ direction. The dispersions near the double Weyl points are linear in the *z* direction. These calculated band structures are proof that the double Weyl points persist in the band structure of the C_3_ photonic crystal.

**Figure 3:**
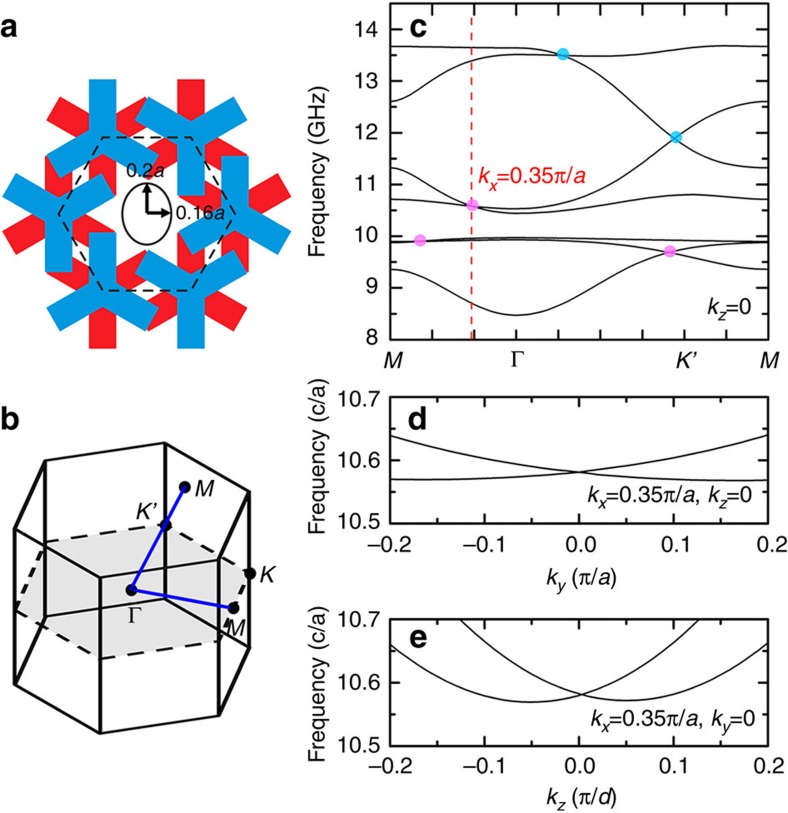
Double Weyl points breaking into single Weyl points due to symmetry breaking. (**a**) Unit cell of the crystal breaking C_3_ symmetry where the PEC cylinder in is replaced by an elliptical cylinder.(**b**) Corresponding Brillouin zone. Blue solid line highlights the path of the **k**-point when calculating the dispersion in (**c**). The upper *M* point, which is related to the lower M point by a reciprocal lattice vector, lies on the Γ−*K*′ direction. (**c**) Band structure in the plane of *k*_*z*_=0. Each of the three double Weyl points at Γ splits into two single Weyl points. Only three of the six single Weyl points are shown (the band crossing points between the second and third bands, the fourth and fifth bands, and the sixth and seventh bands). The other three single Weyl points can be inferred by applying C_2_ rotation about the axis of the elliptical cylinder. (**d**) and (**e**) show the linear dispersions near the Weyl point between the fourth and fifth bands along the *y* and *z* directions, respectively. Interestingly, this tilted Weyl point at 10.6 GHz is in fact a type-II Weyl point having a finite density of states.

If the C_3_ symmetry is broken, two Weyl points with charges of ±1 will separate and each will form a linear dispersion in all three directions. Here we give an example of C_3_ symmetry-broken photonic crystal. Figure 3a shows the top view of the unit cell, which is the same as that in Fig. 1c except that the circular cylinder has been replaced by an elliptical cylinder. Figure 3b,c plot the corresponding Brillouin zone and band structure in the *k*_*z*_=0 plane. Each of the three quadratic dispersive double Weyl points in Fig. 1e at Γ breaks into a pair of single Weyl points with the same topological charge. Three of the six single Weyl points show up as linear dispersive crossing points on the +*k*_*x*_ (along the Γ–*M* direction) or +*k*_*y*_ (along the Γ–*K* direction) axis in Fig. 3c (the degeneracy points between the second and third bands and the fourth and fifth bands along Γ-*M*, and between the sixth and seventh bands along Γ-*K*). These single Weyl points have linear dispersions in all three directions. For example, Fig. 3d,e show the dispersions near the crossing point at (0.35*π*/ *a*, 0, 0) in the *k*_*y*_ and *k*_*z*_ directions, respectively. Another single Weyl point with the same topological charge should lie at (−0.35*π*/ *a*, 0, 0) by applying a C_2_ rotation. Interestingly, these two single Weyl points are actually the so-called type-II Weyl points, which have been discussed very recently in electronic systems^52^ (for example, in WTe_2_). Instead of having point-like equifrequency surfaces as in the case of conventional type-I Weyl points, such type-II Weyl points carry conical equifrequency surfaces at the Weyl point frequencies and their density of states is very different from that of conventional Weyl points. In addition, the two single Weyl points with frequency of 9.7 and 11.9 GHz have shifted from the high symmetry point *K*′ towards the Γ point due to the breaking of C_3_ symmetry. This shifting of Weyl points due to symmetry reduction reflects the robustness of Weyl points in the sense that they cannot be gapped easily and a perturbation simply shifts their positions in **k**-space.

**Figure 4:**
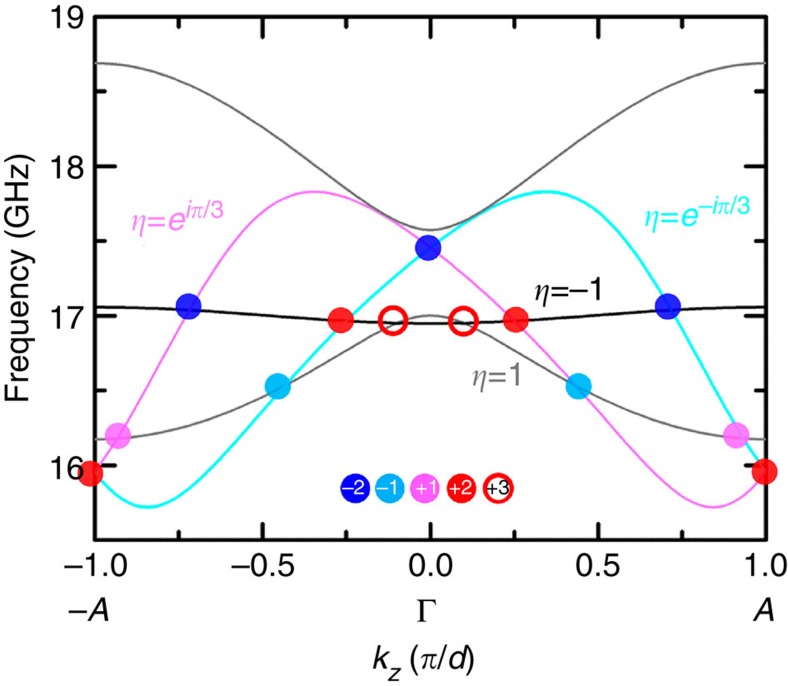
Triple Weyl points along the Γ–*A* direction. Two red open circles highlight the triple Weyl points with a topological charge of +3. Other crossing points (solid circles) between different representations also exist on this C_6_ axis. These crossing points carry topological charges of ±1 or ±2, which are indicated by the colours of the circles. Note that the single Weyl points at ∼16.5 GHz, marked by light blue circles, are type-II Weyl points as well.

In addition, our proposed structure in Fig. 1c also possesses triple Weyl points with a topological charge of 3, each of which is the superposition of three single Weyl points with the same topological charge. Figure 4 plots the band structure along the Γ–*A* direction (the C_6_ axis in our system) in the higher frequency range (>15 GHz) where the bands carrying different C_6_ rotational eigenvalues (*η*) are plotted in different colours. The black band (*η*=−1) and the lower grey band (*η*=+1) cross twice near *k*_*z*_=±0.1*π*/*d* at the frequency of 16.95 GHz. These two crossing points, marked by red open circles in Fig. 4, are triple Weyl points, whose charges are all calculated to be 3. This is consistent with the rotational eigenvalue argument that triple Weyl points emerge when two modes with opposite rotational eigenvalues cross each other along the C_6_ axis in the reciprocal space. Charges of **±**3 are the highest possible topological charges protected by rotational symmetry^50^ and the degeneracy of the three Weyl points is protected by C_6_ symmetry. Note that C_3_ symmetry and time-reversal symmetry cannot protect a triple Weyl point. This is because the two original intersecting bands with opposite C_6_ rotational eigenvalues of −1 and +1 will carry the same representation if the symmetry is reduced to C_3_ and the intersecting bands will have avoided crossing along the Γ–*A* direction (the triple Weyl point splits). In addition to triple Weyl points, there are other crossing points on this C_6_ axis. They are single and double Weyl points with their charges indicated by colour circles. Note that the single Weyl points near 16.5 GHz, marked by light blue circles, are type-II Weyl points. Such exotic topological features come naturally with the structure shown in Fig. 1.

The existence of the single Weyl point at 12.25 GHz can be experimentally confirmed by angle-resolved transmission (Supplementary Notes 2 and 3) and its associated robust surface state can be demonstrated by surface transmission (see below). However, the direct detection of the multiple Weyl points in this system is challenging due to the low excitation efficiency of the bulk modes near these nodal points. The multiple Weyl points are protected by rotation symmetry and time-reversal symmetry and they have linear dispersion in one direction (along the rotation axis) and quadratic (for double Weyl) or cubic (for triple Weyl) dispersion in the in-plane directions. The flat in-plane dispersions near the quadratic or cubic point implies that the impedance of these bulk modes should be quite mismatched with the propagating mode in air, making them difficult to excite. On the other hand, the type-II single Weyl point does not have such limitations. For example, the crossing point at 10.6 GHz in Fig. 3c (C_2_ case) is a type-II Weyl point. The two touching bands can in principle be well separated in vicinity of the nodal point by tuning the geometric parameters of the structure, which will make the type-II point experimentally detectable. In this work, our motivation is to experimentally confirm the robust surface state in the 2D band gap for fixed *k*_*z*_. We choose to realize the photonic crystal with C_6_ symmetry in our experiment, as the higher symmetry ensures a wider bulk gap, which facilitates surface state detection.

### Chern number of the 2D subsystem with a fixed *k*_*z*_

Since the crystal has translational symmetry along the *z* direction, *k*_*z*_ is a good quantum number as long as the translational symmetry is preserved. If we fix a *k*_*z*_ value and consider the dispersion and transport in the *x*-*y* plane, the 2D subsystem would have a 2D band structure in a constant *k*_*z*_ plane (the grey translucent plane in Fig. 1d). *k*_*z*_ is then a parameter that characterizes this 2D system and the Chern number of a nondegenerate 2D band for a fixed *k*_*z*_ is well defined. For example, Fig. 1f shows the bulk band structure in the *k*_*x*_–*k*_*y*_ plane when *k*_*z*_=0.05*π*/*d*. Unlike the band structure of *k*_*z*_=0, the band degeneracies at 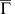 and 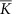 are lifted at nonzero values of *k*_*z*_, opening band gaps so that all the bands for *k*_*z*_=0.05*π*/*d* are separated, and hence their Chern numbers are well defined. We calculated the Chern numbers by analysing the rotational eigenvalues at high symmetry **k**-points^53^. Specifically, we have 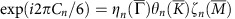, where *C*_*n*_, *η*_*n*_, *θ*_*n*_ and *ζ*_*n*_ are the Chern number, and the C_6_, C_3_ and C_2_ rotational eigenvalues of the *n*th band, respectively. The corresponding Chern numbers are labelled in grey near the bands in Fig. 1f. We found that a nontrivial band gap with a Chern number of 1 opens near 12.25 GHz, which is also observed in our bulk transmission measurement (Supplementary Figs 3–5). By tuning the parameter *k*_*z*_, the eigenmodes with different representations or rotational eigenvalues can swap positions, leading to a change in band Chern numbers. Figure 1g–i plots the dispersion along the *k*_*z*_ direction at 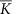, 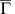 and 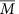, respectively, where the bands with different rotational eigenvalues are plotted in different colours. It can be seen from Fig. 1g,h that several band crossings occur between two bands with different representations. Since these band inversions induce changes in the band Chern number or Berry curvature, the crossing points on these rotational axes should be Weyl points and should carry Berry curvature's charge, which is associated with the jump in Chern numbers. For instance, the black dashed line in Fig. 1j plots the Chern number of the fifth band as a function of parameter *k*_*z*_. It shows several jumps, which coincide with the band inversions occurring at 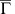 and 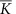. The jumps at *k*_*z*_=0 and *k*_*z*_=*π*/*d* arise from the band inversion at 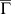 with the fourth band and the inversion at 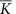 with the sixth band. The jumps at *k*_*z*_=±0.53*π*/*d* and *k*_*z*_=±0.91*π*/*d* are the result of the band inversions at 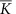 with the fourth band, which are indicated by coloured solid circles in Fig. 1g. The Chern number of the fifth gap, which is the summation of the band Chern numbers below the gap, is illustrated by the red solid line in Fig. 1j. This number is +1 (−1) when *k*_*z*_ is positive (negative), indicating the existence of the surface state propagating clockwise (anticlockwise) in the *x*–*y* plane at the boundary of the crystal.

### Robust surface states on Weyl photonic crystals

**Figure 5:**
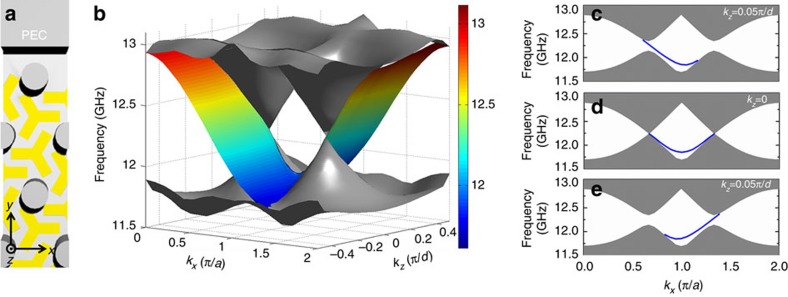
Surface dispersion between the Weyl photonic crystal and the PEC. (**a**) Configuration of the Weyl photonic crystal bounded by a PEC slab. (**b**) Calculated surface dispersion in the surface Brillouin zone. For clarity, only half of the Brillouin zone (*k*_*z*_∈[−0.5 *π*/*d*,0.5 *π*/*d*]) is plotted. Projected bulk states are plotted in grey, while the surface states are plotted in colour. The two linear cones of bulk states lying at the *k*_*z*_=0 line are due to the two Weyl points at K and *K*′ (where the 5th and 6th bands intersect) with a topological charge of −1. The two Weyl points at *H* and *H*′ with a charge of +1, which lie at the *k*_*z*_=*π* line, are not shown. (**c**–**e**) Surface dispersions at *k*_*z*_=−0.05*π*/*d*, *k*_*z*_=0 and *k*_*z*_=0.05*π*/*d*, respectively, where the blue lines denote the surface states.

Owing to the topological charges of Weyl points, the existence of surface states connecting the Weyl points with opposite topological charges is guaranteed by the bulk-surface correspondence^31^. To study the properties of the boundary modes, we consider a Weyl photonic crystal truncated in the *y* direction, bounded by a PEC slab, as illustrated in Fig. 5a. Periodic boundary conditions are applied in both the *x* and *z* directions in our simulation. Calculated surface dispersions near 12.25 GHz are shown in Fig. 5b. Surface states are plotted in colour, while the projected bulk states are plotted in grey. Only half of the Brillouin zone (*k*_*z*_∈[−0.5*π*/*d*, 0.5*π*/*d*]) is shown for clarity. The Weyl points at *K* and *K*′ with frequency of 12.25 GHz are projected onto (*k*_*x*_, *k*_*z*_)=(2*π*/3*a*, 0) and (4*π*/3*a*, 0), respectively. Each of these two Weyl points carries a topological charge of −1. Two linearly dispersive cones of projected bulk bands are formed near these points. Two sheets of surface states, which are connected to one of the two Weyl points, are found in the positive *k*_*z*_ region and the negative *k*_*z*_ region, where the colours indicate the frequency of surface states. The surface states with positive *k*_*z*_ always have positive group velocity (that is, in the +*x* direction), which is consistent with the *k*_*z*_-Chern number in Fig. 1j; the surface states with negative *k*_*z*_ have negative group velocity. When we treat *k*_*z*_ as an additional parameter of the 2D subsystem, Weyl points can be viewed as phase transition points where the bands in a *k*_*z*_ plane change their Chern numbers along with *k*_*z*_. This can be seen by cutting three *k*_*z*_ slices (*k*_*z*_=−0.05*π*/*d*, 0, 0.05*π*/*d*) in the surface dispersion, as shown in Fig. 5c–e. When *k*_*z*_=−0.05*π*/*d*, the gap Chern number between the fifth and sixth bands is −1. The subsystem has an anticlockwise (in the *xy* plane) surface state, as shown by the blue line in Fig. 5c. As *k*_*z*_ increases to 0 (Fig. 5d), the fifth and sixth bands touch at *K* and *K*′ and the surface states are symmetric about *k*_*x*_ as required by time-reversal symmetry. As *k*_*z*_ increases further to 0.05*π*/*d* (Fig. 5e), the 2D band gap reopens with a gap Chern number of 1 and the group velocity of surface states changes direction (now clockwise). Furthermore, the surface dispersion in vicinity of the projection of Weyl point (Supplementary Fig. 6) forms a helicoid^54^ with its winding direction determined by the sign of the topological charge. In addition, two other Weyl points at *H* and *H*′ lie at (2*π*/3*a*, *π*/*d*) and (4*π*/3*a*, *π*/*d*) of the surface Brillouin zone, which are not shown in Fig. 5b. These Weyl points carry a topological charge of +1. Due to the band dispersion along the *y* direction, these two Weyl points are immersed in the projected band of the bulk state in the surface Brillouin zone. The sheets of surface states with positive and negative *k*_*z*_ will eventually merge into the bulk state.

**Figure 6:**
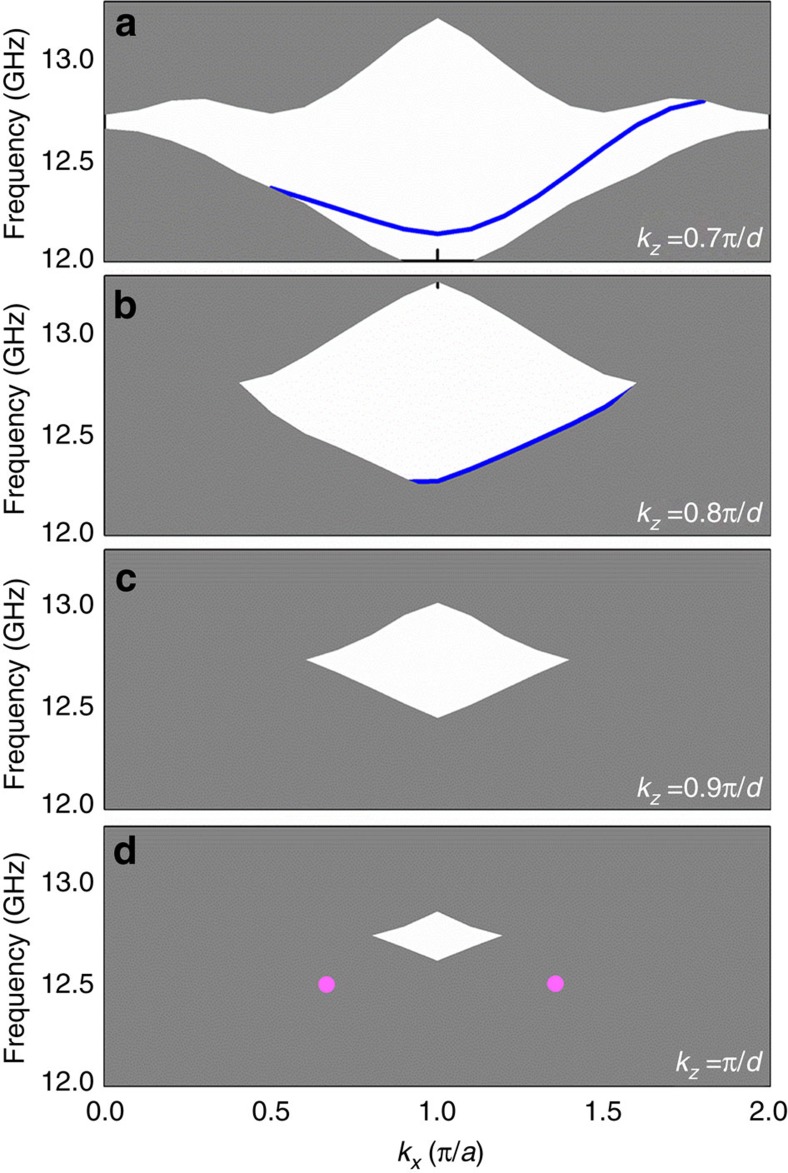
Surface-projected band when (**a**) *k*_*z*_=0.7*π*/*d*, (**b**) *k*_*z*_=0.8*π*/*d*, (**c**) *k*_*z*_=0.9*π*/*d* and (**d**) *k*_*z*_=*π*/*d*. The two pink solid circles highlight the Weyl points between the fourth and fifth bands at *H* and *H*′.

Figure 6a–d shows, respectively, the surface dispersions when *k*_*z*_=0.7*π*/*d*, *k*_*z*_=0.8*π*/*d*, *k*_*z*_=0.9*π*/*d* and *k*_*z*_=*π*/*d*. The complete band gap closes as *k*_*z*_ increases and the surface band (blue) gradually blend into the lower projected bulk band. The two Weyl points at *H* and *H*′ are masked by the other bulk modes along the *y* direction (see the two pink circles in Fig. 6d). Therefore, the sheets of surface states, which should connect the *K* (*K*′) and *H* (*H*′) points of opposite topological charges, will blend into the projected bulk bands following the Weyl points.

**Figure 7:**
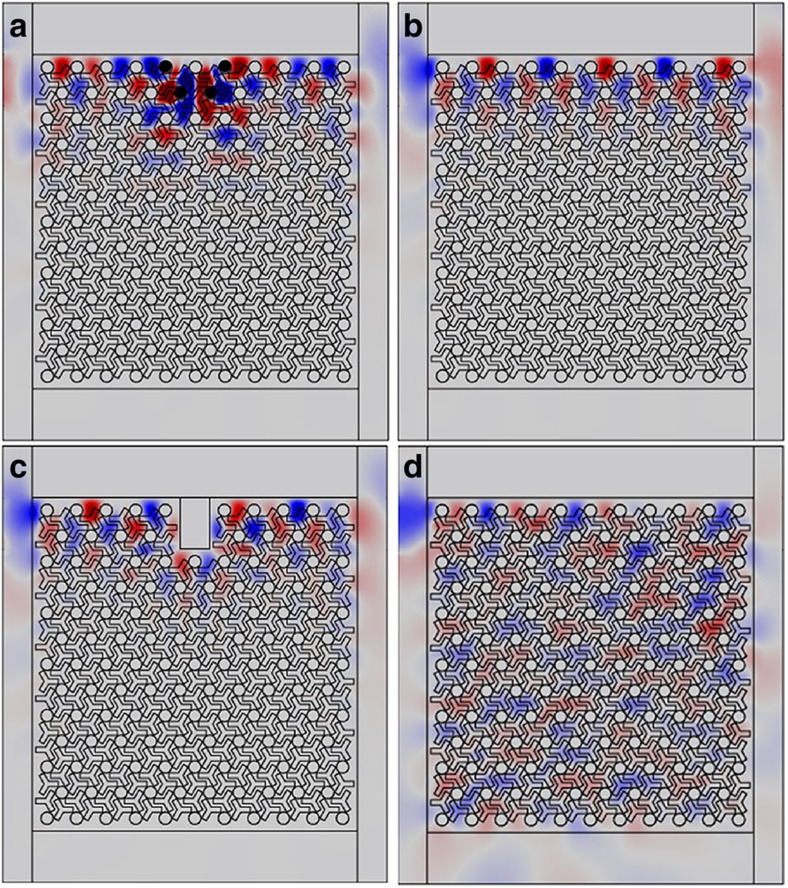
Robust surface states between the Weyl photonic crystal and the PEC. For a fixed nonzero *k*_*z*_, the surface only supports surface states in one direction. Hence the surface states are robust against scattering that preserves *k*_*z*_. (**a**) Simulated *E*_*z*_ field pattern when four PEC rods (shown as black solid circles) near the surface are removed. Electromagnetic (EM) waves with *k*_*z*_=0.3*π*/*d* and frequency of 12.6 GHz hit the photonic crystal from the left. (**b**) Field pattern for the case without defect. (**c**) Field pattern in the presence of a PEC bar. Since both kinds of defects in **a** and **c** are periodic in the *z* direction and *k*_*z*_ is conserved, EM waves can pass through or wrap around the defects without backscattering. (**d**) Field pattern when EM waves with frequency of 11.5 GHz hit the photonic crystal. Wave with frequency outside the band gap can propagate in the bulk crystal. In **a**–**d**, the upper surface of the Weyl photonic crystal is bounded by PEC while the other three surfaces (left, right and bottom) are surrounded by air where EM wave can propagate.

One characteristic property of these chiral surface states is the robustness against the *k*_*z*_-preserving scattering. This can be confirmed by introducing defects, such as removing four PEC rods near the surface of the Weyl photonic crystal (the missing rods shown as black solid circles in Fig. 7a). As these line defects do not disrupt periodicity in the *z* direction, *k*_*z*_ is preserved. Transmission simulations were carried out with an electromagnetic wave of 12.6 GHz impinging on the photonic crystal from the left with *k*_*z*_=0.3*π*/*d*. *k*_*z*_ is set by controlling the phase distribution of the external current source. The surface only supports a rightward surface mode for a positive value of *k*_*z*_=0.3*π*/*d* and hence the electromagnetic wave should be able to pass through these defects without backscattering, as confirmed by numerical simulation in Fig. 7a. The figure displays the E_z_ field pattern, which shows the propagation of a wave confined to the edge that can transport energy to the right through the defects marked by the black dots. The upper surface of the photonic crystal is bounded by PEC, while the other three surfaces (left, right and bottom) are surrounded by air where electromagnetic wave can leak out. Therefore, a large amount of the electromagnetic wave will propagate into air after passing through the upper-right corner. For the sake of comparison, Fig. 7b shows the field pattern in the case without defects. Figure 7c gives the simulated field pattern of another example, with a PEC bar inserted into the bulk of the photonic crystal. An electromagnetic wave of 12.6 GHz impinging on the photonic crystal from the left can wrap around this defect and keep moving rightward. The field patterns in Fig. 7a–c show that the wave propagation is indeed confined to the edge as expected for an edge mode with the frequency falling within the bulk band gap. In comparison, if the frequency of the incident electromagnetic wave falls outside of the nontrivial band gap, the wave would propagate into the bulk crystal as shown in Fig. 7d which simulates the case with a frequency of 11.5 GHz and without defects.

We note that the surface state's robustness against *k*_*z*_-preserving backscattering is not a generic feature of Weyl crystals. The topological charge only guarantees the existence of surface states connecting the projections of Weyl points with non-vanishing charges on the surface Brillouin zone. The *k*_*z*_-preserved one-way surface state is protected by the nonzero Chern number on a fixed *k*_*z*_ plane, as plotted in Fig. 1j. The *k*_*z*_-Chern number can be nonzero only if the in-plane mirror symmetry (*k*_*x*_–*k*_*y*_ plane) is broken. The surface transmission associated with zero *k*_*z*_-Chern number should be sensitive to the defect because the surface supports both forward and backward propagating surface states for the fixed-*k*_*z*_. However, for the chiral interlayer coupling system, the nonzero *k*_*z*_-Chern number indicates that the surface only supports the surface state propagating in one direction (clockwise or anticlockwise) which is backscattering-immune.

**Figure 8:**
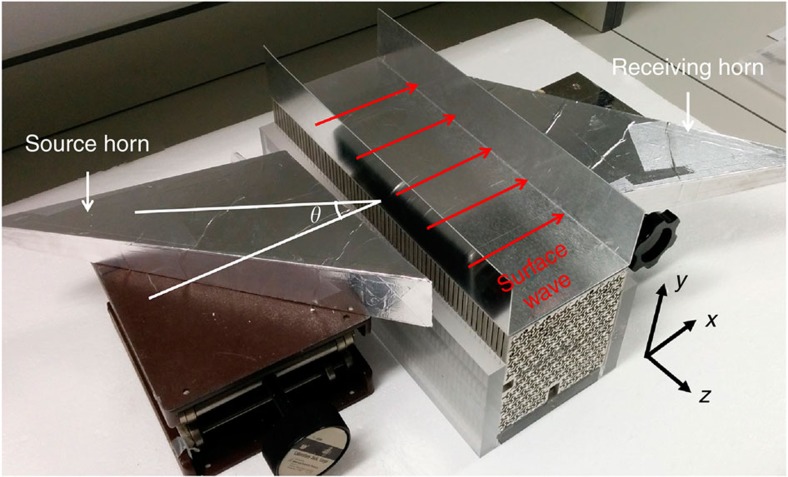
Experimental set-up for surface transmission measurements. Two sectoral horns with sample tilted angles of 35° or 50° are used to emit and receive microwave. The sample has 11, 13 and 60 periods in the *x*, *y* and *z* directions.

**Figure 9:**
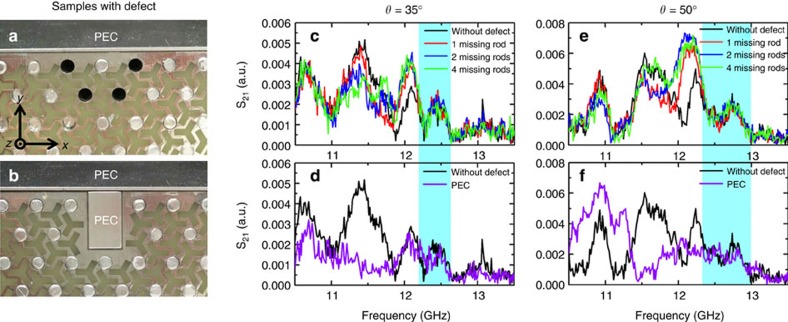
Measured surface transmissions when different kinds of defects are introduced near the surface. (**a**,**b**) Photograph of the samples with defects. (**c**,**d**) Measured transmission spectra for two kinds of defects when the incident angle *θ*=35° (*k*_*z*_=0.2581*π*/*d* for 12.5 GHz). (**e**), (**f**) Results for *θ*=50° (*k*_*z*_=0.3447*π*/*d* for 12.5 GHz). Cyan boxes in **c**–**f** highlight the frequency region of *k*_*z*_-preserved one-way surface states where no obvious backscattering is introduced by the two kinds of defects. Note that the frequency region of robust transport for *θ*=50° is wider than that for *θ*=35°. This is consistent with the fact that the nontrivial band gap in the reduced 2D Brillouin zone is wider for a larger *k*_*z*_.

In electronic systems, the angle-resolved photoemission spectroscopy technique has been used to detect the surface Fermi arcs of Weyl semimetals^35^,^36^. Since vacuum is transparent for photons and can be considered as gapped for bound electrons, illuminating the surface with light can excite the surface electron wave localized between vacuum and a Weyl semimetal (Here for simplicity, we assume the surface is the *xz* plane). On the other hand, for photonic systems, the chiral surface states exist between the Weyl photonic crystal and another opaque material such as the PEC in our measurement. And we cannot excite the surface electromagnetic state by impinging an electromagnetic wave on the PEC. Hence the angle-resolved technique commonly used for electronic systems cannot be applied to extract the surface momentum (*k*_*x*_) of the chiral surface state. However, we can still observe the chiral surface state without having explicit information about the *k*_*x*_ component while the *k*_*z*_ component can be tuned by controlling the angle of the incident electromagnetic wave. To confirm the existence of the chiral surface state, we first measured the transmission of the surface without any defect in the surface region (Methods). The experimental set-up is shown in Fig. 8. The black curve in Fig. 9c plots the result for an incident angle of 35° (relative to the *xoy* plane). The 2D band structures for a fixed *k*_*z*_ have band gaps as confirmed experimentally (Supplementary Note 2) and the bulk cannot transmit in the frequency range of the band gaps. The high surface transmission in the frequency region of the bulk band gap (∼12 to 12.7 GHz for *θ*=35°) implies the existence of surface states although *k*_*x*_ cannot be determined explicitly.

To test experimentally, the robustness of the surface state, samples with these defects were fabricated. Figure 9a,b shows photographs of the samples. An electromagnetic wave impinged on the left of a sample at a specific incident angle *θ*. Figure 9c shows the results for the sample with missing rods hit by an electromagnetic wave at an incident angle of 35° (*k*_*z*_=0.26*π*/*d* for 12.5 GHz). We measured transmittances for the cases with one, two and four missing rods (red, blue and green curves in Fig. 9c). Compared with the transmittance of the sample without defects (black curve), we found that the spectra overlap with each other in the frequency range from 12.2 to 12.65 GHz (light cyan box), where the system has a band gap with a nontrivial Chern number (see also the transmission spectra with log scale in Supplementary Fig. 7). The results indicate that the transmission is almost the same with and without the missing rods and removing the aluminium rods did not introduce backscattering in the nontrivial band gap with a nonzero *k*_*z*_. However, the bandwidth of robust transport is somewhat narrower than the band gap predicted by the bulk band structure. This is due to the finite beam width of the incident wave packet, which leads to the spread of the Fourier components around *k*_*z*_=2*πf* sin *θ*/*c*. The purple curve in Fig. 9d plots the measured transmittance when an aluminium bar was inserted into the crystal as shown in Fig. 9b. Robust transport was also observed from 12.2 to 12.65 GHz where the system has a nontrivial gap. Within this frequency range, the transmittance is almost the same with and without the interrupting metal bar. In the frequency region below 12.2 GHz, noticeable differences exist. The transmissions with defect are greater than the transmission without defect at some frequencies (for example, 11.9–12.2 GHz in Fig. 9c). The reason is that in the frequency region of the passing band, most of the microwave propagates in the bulk crystal (Fig. 7d) and the defect can perturb the field pattern at the right end of the surface where the surface transmission was measured. The defect near the surface will affect the signal received by the receiving horn but not necessarily block the signal because this is a complicated multiple reflection process when the waves propagate inside the crystal. The transmission in the presence of the defect can be either larger or smaller than the one without the defect (black curve) at different frequencies. This is also confirmed by the calculated transmission (Supplementary Note 4; Supplementary Fig. 8). In the frequency region >12.65 GHz, the transmission difference is not obvious due to the weak signal (low signal to noise ratio). However, we can still see the difference at some particular frequencies. For example, the transmission peak at 13.06 GHz for the black curve in Fig. 9c,d. At this frequency, the transmissions with defects (coloured curves) decrease in different extent which implies that the defects do affect the transmission outside the nontrivial band gap.

Due to bulk-surface correspondence, the surface between Weyl photonic crystal and PEC supports *k*_*z*_-preserved one-way surface states as long as the crystal has a complete band gap characterized by nonzero Chern numbers at that value of *k*_*z*_. Figure 1j shows the nonzero Chern number of the 5th gap (around 12.5 GHz) when *k*_*z*_ is not equal to 0 or *π*/*d*. This nontrivial complete band gap remains open when *k*_*z*_ is smaller than 0.7*π*/*d* although the gap width will change as *k*_*z*_ increases (Supplementary Fig. 4c,h). Since the wave number in vacuum (maximal *k*_*z*_) is 0.45*π*/*d* for 12.5 GHz, robust surface transmission should occur for all the incident angle except 0°. The gap width will change along with the incident angle. When *k*_*z*_=0, the 5th and 6th bands touch at *K* and *K*′. The nontrivial band gap opens for nonzero *k*_*z*_. And the gap width increases as *k*_*z*_ increases when *k*_*z*_ is smaller than 0.4*π*/*d*. From Supplementary Fig. 4, it can be seen that incident angle of 50° has a bigger gap than that of 35°. To confirm this, Fig. 9e,f show the measured transmittances for the incident angle of *θ*=50° (*k*_*z*_=0.3447*π*/*d* for 12.5 GHz). Similar robust transmissions were observed from 12.3 to 13 GHz (see the light cyan box), which is broader than the range for the case of *θ*=35°. This is consistent with Fig. 1g–i, which show that the gap width of this nontrivial band gap increases as *k*_*z*_ increases when *k*_*z*_<0.4*π*/*d*. Again, this gap width is narrower than the expected range (from 11.8 to 13 GHz) due to the finite beam width. Many other systems such as 2D photonic quantum (spin) Hall systems^5^,^6^,^7^,^8^,^9^,^10^,^11^,^12^,^13^,^14^,^15^,^16^,^17^,^18^,^19^,^20^,^21^,^22^ are immune to backscattering. The Weyl photonic system in this study is a 3D time-reversal-invariant system possessing immunity against *k*_*z*_-preserving backscattering.

## Discussion

We designed and fabricated a microwave Weyl photonic crystal that carries both single and multiple Weyl points. The double Weyl points in our system are protected by C_3_ and time-reversal symmetries while the triple Weyl points are protected by C_6_ symmetry. The associated topologically protected chiral surface states between the Weyl photonic crystal and the PEC were also measured and demonstrated to be robust against *k*_*z*_-preserving scattering. For a fixed *k*_*z*_, our Weyl photonic crystal can be regarded as a 2D subsystem where synthetic gauge flux is introduced by chiral interlayer coupling. Adding in-plane inversion (C_2_) symmetry breaking would enable our system to emulate all the regimes in the phase diagram of the Haldane model^2^. Double and triple Weyl points imply a larger Berry flux than single Weyl points in the neighbourhood, and can induce a stronger anomalous velocity effect. This enhanced effect manifests in the deflection of the path of a wave package propagating inside the bulk material.

## Methods

### Tight-binding model of AA-stacked honeycomb lattice

To see how the tight-binding model guides us to the structure presented in Fig. 1, we first consider a single-layer honeycomb lattice whose Bloch Hamiltonian can be written as


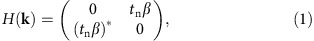


where 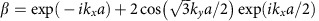, and *a* is the distance between the two sublattices, *t*_n_ is the nearest neighbour hopping and (*k*_*x*_, *k*_*y*_) is the Bloch wave vector. By applying the *k·p* method, one can obtain its effective Hamiltonian (Dirac form) near *K* point (*k*_*x*_=0, 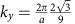)


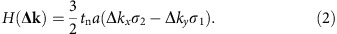


where *σ*_*i*_ is the Pauli matrix and Δ**k**=(Δ*k*_*x*_, Δ*k*_*y*_) is a small **k** near *K* point.

We then consider the effect introduced by the interlayer coupling of a multilayer system. Supplementary Figure 1 depicts the unit cell of an AA-stacked honeycomb lattice where hopping is nonzero only between sites connected by the solid lines. The layer distance is *d*. Blue lines highlight the chiral interlayer coupling with a real hopping coefficient of *t*_c_. After some algebra, one can obtain its Bloch Hamiltonian


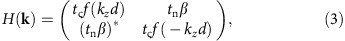


where 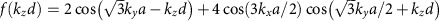. Expanding the Hamiltonian near *K* point (*k*_*x*_=0, 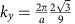, *k*_*z*_=0), we have


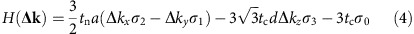


which implies that it is a Weyl point with charge of −1. In this sense, the chiral interlayer coupling between single-layer Dirac systems introduces an additional Δ*k*_*z*_*σ*_3_ term and the multilayer system forms a Weyl point at *K*. The tight binding model results give us a guide to the type of connectivity in real space that can give rise to Weyl points in the momentum space. This is of course just the starting point and the realization of such chiral coupling in real photonic crystal depends on experience and intuition gained in working with photonic crystals and tedious iterative fine tuning between simulations and design is required. The actual band structure of the real system must of course be calculated using a full-wave simulation.

### Detailed geometry of the Weyl photonic crystal

Our Weyl photonic crystal can be realized by stacking the PCBs in the *z* direction, with copper cladding on both sides and a hexagonal array of metal cylinders piercing through the stack. The PCBs were 0.43*a* thick and they were spaced 0.11*a* apart in the *z* direction, where *a*=1 cm is the lattice constant of the hexagonal array. The dielectric constant of the FR4 substrate (yellow in Fig. 1b) was 4.8 and the thickness of the deposited copper layer (shown in grey and assumed to be PEC in our simulation) was 40 μm. This structure had a 3D hexagonal lattice with in-plane lattice constant *a* and out-of-plane lattice constant *d*=0.54*a*. Figure 1d shows its 3D Brillouin zone. Interlayer coupling are introduced by etching Y-shaped slots on the top and bottom copper layers. Figure 1c shows the top view of the unit cell (dashed hexagon), where the Y slots on the top and bottom surfaces are shown in blue and red, respectively. It can be seen that the slots form a chiral pattern and break all the mirror symmetries and inversion symmetry. The metallic components (rods and copper layers) in the photonic crystal form a connected metallic network, which leads to a cutoff frequency of 9.23 GHz for the bulk modes.

### Double Weyl points protected by symmetries

To prove that C_3_ and time-reversal symmetries can protect double Weyl points at time-reversal-invariant **k**-points, we derive an effective theory following the algebra described in ref. 50. Suppose that a time-reversal-invariant **k**-point Λ_0_ lies on a C_3_-invariant line (for example, Γ point or A point lying on the *k*_*z*_ axis), the effective Hamiltonian near this point can be written as


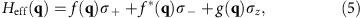


where **q** denotes the in-plane **k**-vector deviating from Λ_0_.

We further assume that two bands crossing at Λ_0_ are linked by time-reversal symmetry, which is the case shown in Fig. 2. Then the C_3_ eigenvalues of the two bands cannot be 1, otherwise the bands would have avoided crossing. If the first band's C_3_ eigenvalue is exp(*i*2*πp*/3) (*p*=±1), then the second band's should be exp(−*i*2*πp*/3) by applying time-reversal. Note that the basis in the above Hamiltonian are the two eigen states at Λ_0_ with different representations. (1, 0)^*T*^ represents the eigen state with the C_3_ eigenvalue of exp(*i*2*πp*/3), while (0, 1)^*T*^ represents the eigen state with the C_3_ eigenvalue of exp(−*i*2*πp*/3). In this basis, the matrix representations of C_3_ rotation and time-reversal symmetry are


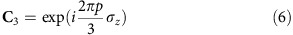


and


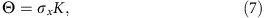


where *K* represents the complex conjugation. Due to the C_3_ symmetry, we have


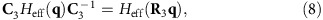


where **R**_3_(*q*_+_, *q*_−_)=(*q*_+_*e*^*i*2*π*/3^, *q*_−_*e*^−*i*2*π*/3^) and *q*_±_=*q*_*x*_±*iq*_*y*_.

Substituting equation (6) into equation (8) yields


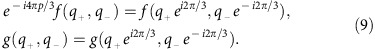


We can expand *f*(*q*_+_, *q*_−_) near Λ_0_ as follows:


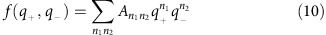


Equation (9) requires that


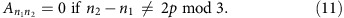


On the other hand, due to time-reversal symmetry, we have


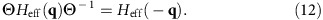


Substituting equations (5) and (7) into equation (12) gives


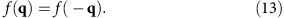


According to equation (10), the linear terms (*A*_10_ and *A*_01_) must vanish. Combining these with equation (11) and taking the smallest *n*_1_+*n*_2_ reveals that the crossing point at Λ_0_ should carry a topological charge of 2*p* (ref. 50).

### Experimental set-up

All the samples used in bulk and surface measurement consisted of 60 PCBs (about 32.4 cm thick) stacked in the *z* direction. The 60 PCBs were supported by two plexiglass slabs and pierced through by aluminium rods. Supplementary Figure 3 shows the experimental set-up for bulk transmission measurement. electromagnetic waves were emitted from the left horn antenna with the electric field polarized in the *xz* plane, and received by the right antenna with the same polarization. When an electromagnetic wave impinged on a sample at an incident angle *θ* and frequency *f* as shown in Supplementary Fig. 4b, all the bulk modes with *k*_*y*_=0 and *k*_*z*_=2*πf* sin*θ*/*c* were excited as shown by the blue dashed line in Supplementary Fig. 4a. *θ* could be adjusted by rotating the sample and one can scan the bulk modes with different *k*_*z*_. Although the samples used in our bulk transmission measurement had only four or five periods in the propagation direction (*x* direction), they still enabled observing the transmission contrast between the bulk band gap and the passing band (for example, the transmission below and above 10.3 GHz in Supplementary Fig. 4e,j). It was impractical to employ more periods in the *x* direction as a thicker sample in *x* would mandate corresponding increases in *y* and *z*, which would make the sample unwieldy in the transmission measurement and the signal reaching the backside would also be weak.

Figure 8 shows the set-up for surface transmission measurement. The sample used in surface measurement had 11 and 13 periods in the *x* and *y* directions, which were large enough to decouple the surface states on the top and bottom surfaces. The sample was supported by plexiglass slabs on two sides and capped by an aluminium slab with two walls. The slab was assumed to be PEC in the microwave region. The two walls of the aluminium slab served to block the wave that was propagating in air from the source horn to the receiving horn. The zoomed-in areas near the surface are illustrated in Fig. 9a,b. To enhance the coupling between the input (output) wave and the surface state, a pair of sectoral horns with tilted angles of 35° or 50° were used to emit and receive microwave. Figure 9a shows the configuration of four missing rods. For the case of two missing rods, only the two rods closest to the surface were removed. The measured results in Fig. 9c–f are plotted in linear scale (see also the results in log scale in the Supplementary Fig. 7).

All simulations were performed using the commercial solver package COMSOL Multiphysics v.4.4 (COMSOL Inc., 2013). In the simulation shown in Fig. 5, periodic boundary conditions were applied in both the *x* and *z* directions in our simulation. In the simulations of Fig. 7, photonic crystals with infinite height were considered as periodic boundary conditions were used in the *z* direction.

### Data availability

The data that support the findings of this study are available from the corresponding author on request.

## Additional information

**How to cite this article:** Chen, W.-J. *et al*. Photonic crystals possessing multiple Weyl points and the experimental observation of robust surface states. *Nat. Commun.* **7,** 13038 doi: 10.1038/ncomms13038 (2016).

## Supplementary Material

Supplementary Information Supplementary Figures 1-8 and Supplementary Notes 1-4
