# Supplementary material for: Photonic crystals possessing multiple Weyl points and the experimental observation of robust surface states
Source: Nat Commun. 2016 Oct 5;7:13038. doi: 10.1038/ncomms13038 (PMC5059475; doi:10.1038/ncomms13038)
Supplement: Supplementary Information — Supplementary Figures 1-8 and Supplementary Notes 1-4 [file ncomms13038-s1.pdf]

Supplementary Figures

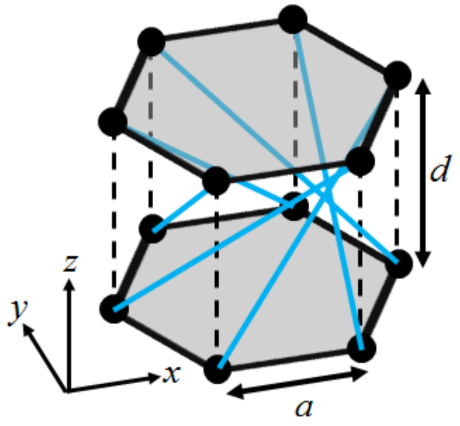

Supplementary Figure 1 | Tight-binding model of an AA-stacked honeycomb lattice. The hoppings are nonzero only between sites connected by the solid lines. a is the distance between two sublattices and d is the layer distance. Black (blue) solid lines highlight the intralayer (interlayer) coupling with a real hopping coefficient of t_n (t_c).

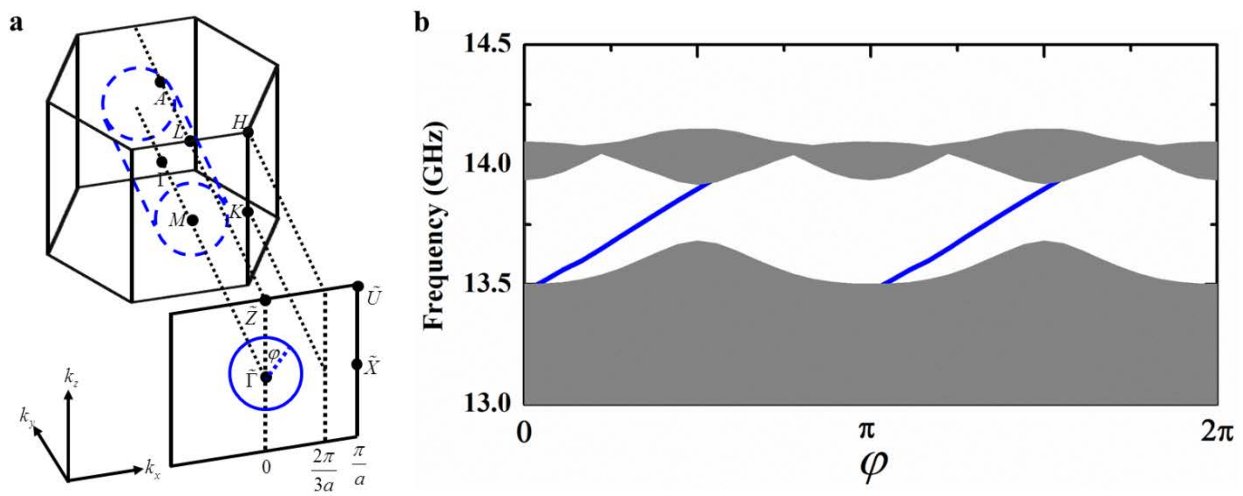

Supplementary Figure 2 | Surface dispersion on a closed loop encircling the double Weyl points. (a) Bulk Brillouin zone (upper) and surface Brillouin zone (lower). Blue solid curve (with radius of $0.5\pi / a$) highlights the path where we calculate the surface dispersion. Blue dashed lines in bulk Brillouin zone highlight the surface (a tube) projected onto the blue circle. (b) Surface dispersion on the blue circle as a function of angular coordinate φ. Two gapless surface states imply that the total charge of 2 is encircled by the tube in bulk Brillouin zone.

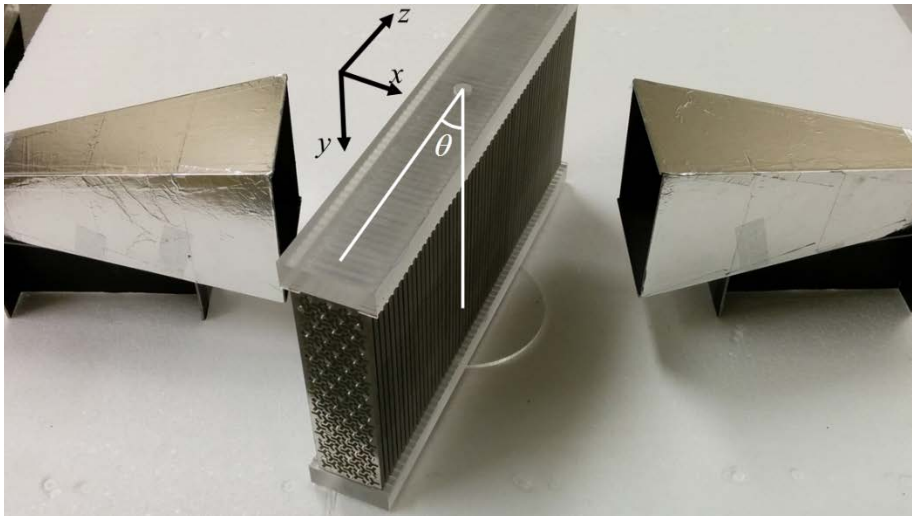

Supplementary Figure 3 | Experimental setup for bulk transmission measurements. EM waves are emitted and received by two horn antennas with electric field lying in the xz plane. By changing the incident angle θ, bulk states with different k_z=$2\pi f sin\theta/c$ can be excited.

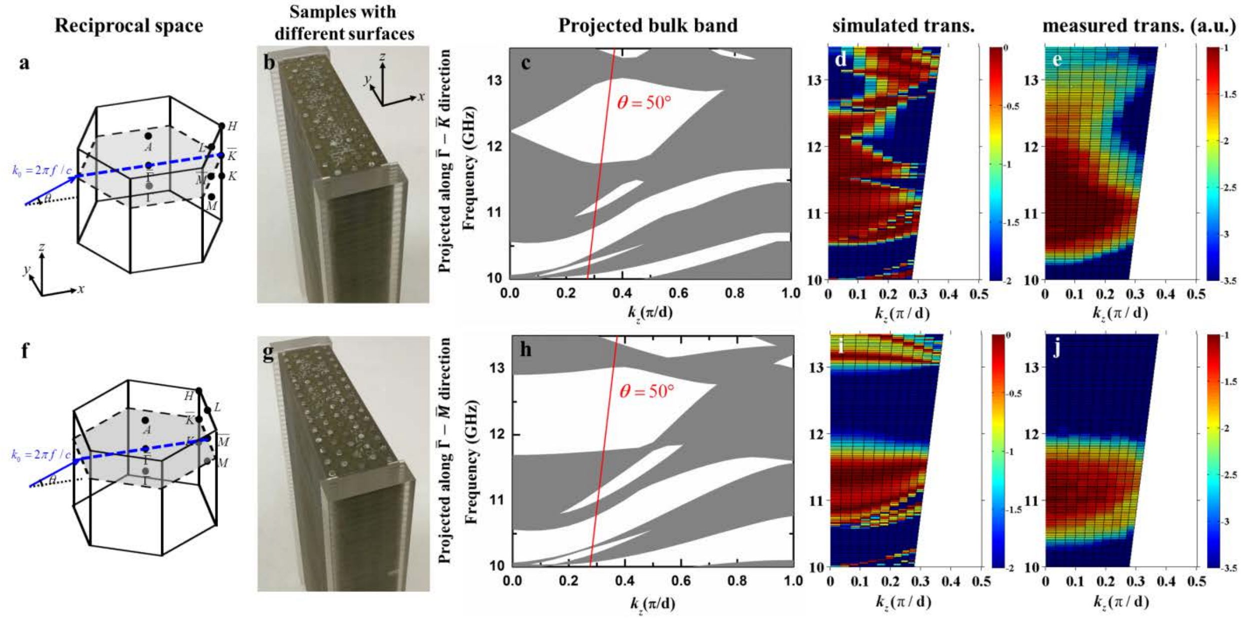

Supplementary Figure 4 | Projected band structure and bulk transmission. (a) Reciprocal space corresponding to the sample shown in (b). (b) Photograph of the sample with surface perpendicular to the Γ−K direction. Blue arrow in (a) indicates the incident wave vector in the xoz plane with tilted angle θ. The bulk modes lying at the blue dashed line, which is parallel to the $\overline{\Gamma}-\overline{K}$ direction, can be excited. (c) Bulk band projected along the $\overline{\Gamma}-\overline{K}$ direction as a function of k_z. The red line depicts the k_z corresponding to the maximal incident angle ($\theta = 50°$) in our measurement. (d), (e) simulated and measured transmission spectra along the $\overline{\Gamma}-\overline{K}$ direction. (f) Reciprocal space corresponding to the sample shown in (g). (g) Sample with surface perpendicular to the Γ−M direction. (h) Bulk band projected along the $\overline{\Gamma}-\overline{M}$ direction. (i), (j) simulated and measured transmission spectra along the $\overline{\Gamma}-\overline{M}$ direction.

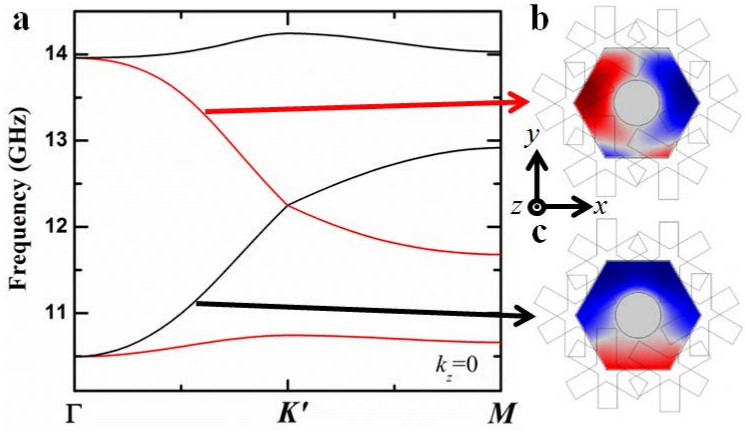

Supplementary Figure 5 | Bulk band structure along $\Gamma - K'$ direction (k_y direction). (a) Bluk band structure. Red lines highlight antisymmetric modes. (b), (c) the E_z field pattern of the antisymmetric and symmetric modes.

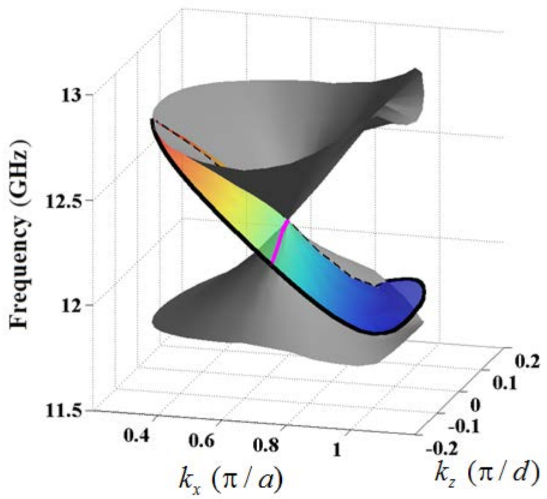

Supplementary Figure 6 | Surface dispersion near the Weyl point at $(k_x, k_z) = (2\pi / 3a, 0)$. Black curve highlights the surface state at a fixed radius of **k**. Magenta curve shows the equifrequency contour at the Weyl frequency.

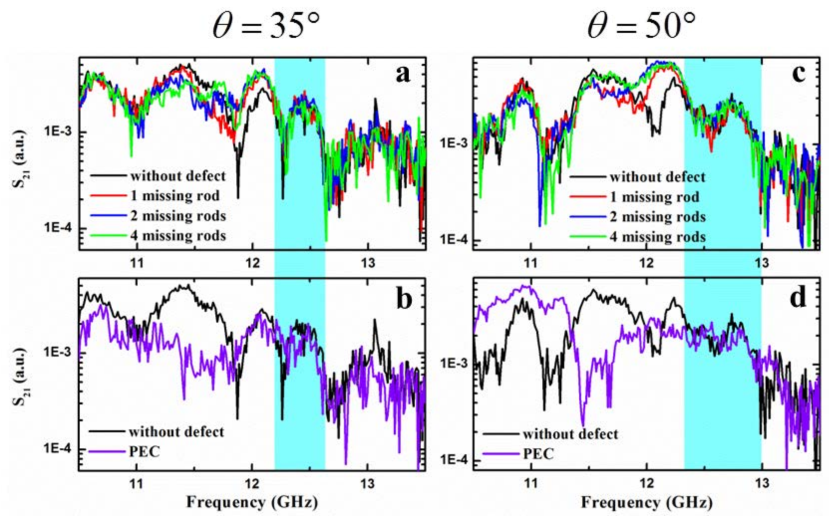

Supplementary Figure 7 | Measured surface transmissions with log scale. (a), (b) The measured transmission spectra for two kinds of defect when the incident angle θ=35° (k_z=0.2581π/d for 12.5GHz). (c), (d) The results for θ=50° (k_z=0.3447π/d for 12.5GHz). Cyan boxes in (a)-(d) highlight the frequency region of k_z-preserved one-way surface states where no obvious backscattering are introduced by the two kinds of defects.

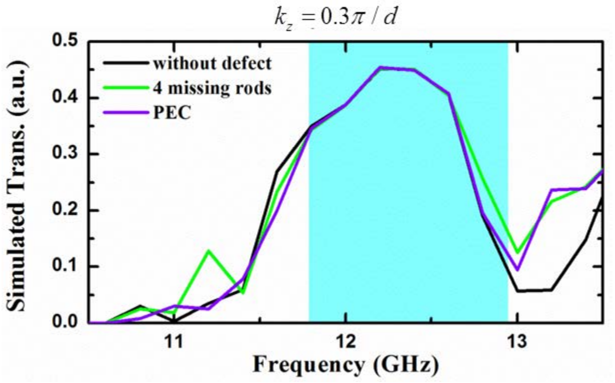

Supplementary Figure 8 | Simulated surface transmissions when k_z=0.3π/d. The transmission is calculated by integrating the electric field strength at the right end of the surface. Cyan box highlights the nontrivial bandgap for k_z=0.3π/d predicted by calculated band structure. Inside the bandgap, the three curves almost overlap with each other indicating robust transport. Outside the bandgap, waves can propagate in the bulk of the crystal. Therefore the transmission with defect (green or violet curve) can be either larger or smaller than the one without defect (black curve) at different frequency.

Supplementary Note 1

The charge of double Weyl points

To see that the charge of double Weyl points is two, one can integrate the Berry flux on a closed surface which encloses the Weyl points. We can also manifest the topological charge of double Weyl point by calculating the surface dispersion in the following mathematical reconstruction. Let us consider a tube in k-space aligned along the y direction with a fixed radius $k_r = 0.5\pi / a$ (rather than k_z) in the Brillouin zone to obtain a 2D subsystem, see the blue dashed tube in Supplementary Fig. 2a. This tube is a torus since it is periodic in k_y direction. The 2D bands on this tube (with a fixed k_r and k_y, k_ϕ are varying) have well-defined Berry curvatures and Chern numbers. If the 2D subsystem has a band gap, and if we terminate the crystal perpendicular to the y-direction, the number of surface states should be equal to the gap Chern number of this 2D gap due to the bulk-edge correspondence. The gap Chern number is related to the Weyl points' charge in the following way. The gap Chern number is the sum of band Chern numbers below that gap. Each band Chern number can be calculated by integrating the Berry flux on the tube or by summing the topological charges enclosed within the tube using the Stokes theorem. Note that a Weyl point with charge of Q will contribute integrated Berry flux of equal magnitude but opposite sign to its upper and lower band.

Now we consider the band gap between the 6th and 7th bands. From Fig. 1i, we know that no Weyl point lies at M. The Berry flux on the tube only comes from the Weyl points at Γ. Since the Weyl point at 10.05 GHz in Fig. 1e contribute opposite charges to the 2nd band and the 3rd band, it does not contribute to the gap Chern number between the 6th and 7th bands. Likewise, the Weyl point at 10.5 GHz does not contribute either. Hence this gap Chern number is only related to the Weyl point at Γ between 6th and 7th bands near 14 GHz and should be the opposite number (+2) of the topological charge of this Weyl points (-2) [and hence contribute +2 to the lower band]. Therefore, if we terminate the crystal in y-direction, there should be two gapless surface states.

Supplementary Figure 2b calculates the surface dispersion with the geometry described in Fig. 5a in the frequency region of the 6th band gap. It is calculated on a closed circle (blue solid curve in Supplementary Fig 2a) in the surface Brillouin zone to be consistent with the 2D subsystem we discussed above. Two gapless surface states (propagating in clockwise direction) indeed exist in this nontrivial band gap, which implies the -2 charge of this double Weyl points.

Supplementary Note 2

Bulk transmission measurements

To experimentally confirm the existence of Weyl points, we measured angle-resolved transmission of the Weyl photonic crystal. Two samples were fabricated, one with the surface normal along the $\overline{\Gamma}-\overline{K}$ direction (as shown in Supplementary Fig. 4b), the other along the $\overline{\Gamma}-\overline{M}$ direction (Supplementary Fig. 4g). Gray areas in Supplementary Fig. 4c are the calculated bulk band projected along $\overline{\Gamma}-\overline{K}$ direction. The red line marks the k_z corresponding to $\theta = 50°$, which is the maximal incident angle in our measurement. From the projected bulk band in Supplementary Fig. 4c, we see that the directional band gap between the fifth and the sixth bands in the $\overline{\Gamma}-\overline{K}$ direction opens at nonzero k_z and becomes larger as k_z increases when $k_z < 0.3\pi / d$. This can be seen by the white region at about 12 GHz, together with the linear dispersion of the Weyl point at K.

Supplementary Figure 4d shows the simulated transmission spectra with different incident angles. We can clearly see the projected linear cones at about 12.25 GHz in the transmission spectra. Apart from this, we found fairly low transmission in the simulation above 12.5 GHz when $k_z < 0.1\pi / d$, even though there are allowed bulk modes in that part of the momentum space according to the projected band. The reason is that projected band only tells whether there are states or not while transmission spectra also include the information of the coupling between modes in the photonic crystal and the source. In this specific case, the incident plane wave cannot excite the antisymmetric mode along $\Gamma - K$ axis. These antisymmetric modes are even under the C_2 rotation along the y axis (the $\Gamma - K$ direction), as shown below in Supplementary Note 3. When k_z increases, these modes become excitable. Besides this mode

coupling issue, another difference is the additional interference fringes in Supplementary Fig. 4d (for example, the low transmission around 11.5GHz and k_z around $0.2\pi / d$). These interference fringes are caused by multiple reflections at the two interfaces between air and photonic crystal since their effective mode impedances are different. Except for the above mentioned two discrepancies, the simulated transmission spectra are similar to the projected bulk band structure.

The corresponding measured transmission is shown in Supplementary Fig. 4e. We can see the band edge around 10.3 GHz and the linear cone around 12.2 GHz. Note that the amplitude of the measured transmission is smaller than the calculated transmission. This is due to the difference between the simulation and experimental setups. In simulation we can collect all the waves transmitting through the photonic crystal, while in our experiment, the horn antennas emit or receive EM wave with finite beam width. The beam will spread out (in both *y*- and *z*-directions) during its propagation in air or the multiple reflection process in the sample. The receiving antenna can only receive part of the transmitted beam, which leads to the overall decrease of the measured transmissions in Supplementary Fig. 4e compared with the simulated result in Supplementary Fig. 4d. We also note that the band edges in the experiment seem a little blurred. In the simulation, the incident wave can be assigned a specified k_z value; while in the experiment, k_z value is chosen by tilting the horn angle relative to the photonic crystal (see Supplementary Fig. 3) and this provides us incident wave with a finite spectrum range of k_z. Hence the measured spectrum loses the fine structures and appears blurred when compared with the simulated spectrum. In addition, due to the fabrication error of the sample and the deviation of the dielectric constant of PCB substrate from the standard value, the lower band edge in measured spectrum shifts 2% from that of simulated spectrum.

In contrast to the $\overline{\Gamma}-\overline{K}$ direction, one finds that the directional band gap along $\overline{\Gamma}-\overline{M}$ direction always opens for $k_z < 0.4\pi / d$. Supplementary Figures 4h and 4i are the corresponding projected bulk band and simulated transmission. The simulated spectrum agrees well with the projected band except that the upper passing band above 13 GHz split into three red zonal regions. These in fact correspond to three peaks in each transmission spectrum for different incident angle, due to the multiple reflections at the two interfaces between photonic crystal and air. We note that the amplitude of these fringes (difference between the peak value and deep value) above 13 GHz in Supplementary Fig. 4i is much larger than those in Supplementary Fig. 4d. This phenomenon indicates that there is a significant mode impedance mismatch between source (in air) and modes of this band. It is natural to imagine that the EM wave will bounce forward and back many times before transmitting through the photonic crystal.

Supplementary Figure 4j shows the corresponding measured transmission spectrum. We can clearly see the lower passing band from 10.3 to 11.8 GHz. However the interference fringes in the upper passing band cannot be observed. This is because at these Fabry-Perot resonant frequencies, EM waves will be bounced between the two interfaces many times and constructively interfere at the transmitted interface. In our measurement, we impinge the incident beam with finite beamwidth onto the finite size crystal. The beam will spread in *y*- and *z*-directions during the multiple reflection process inside the crystal, reach the side boundaries of the sample and leak outside. Hence we can hardly receive any signal considering the finite width of the horn as a receiver. For the same reason, the unapparent interference fringes in Supplementary Fig. 4d cannot be observed in our measured result in Supplementary Fig. 4e.

Together with the transmission spectra along $\overline{\Gamma}-\overline{K}$ direction, we found that a 2D complete band gap opens near the Weyl point frequency of 12.25 GHz in k_x-k_y plane for nonzero

k_z and that the gap width broadens as k_z increases. The calculated nonzero Chern number of this band gap indicates the existence of a chiral surface state, which was confirmed in our surface measurement.

Supplementary Note 3

Antisymmetric bulk modes of the photonic crystal

In the discussion concerning bulk transmission, we mentioned that the low transmission in Supplementary Fig. 4d, when frequency is bigger than 12.5 GHz and k_z<0.1, is due to the symmetry-forbidden excitation of antisymmetric mode by external plane waves. Supplementary Figure 5a shows the band structure along the $\Gamma - K'$ direction (k_y direction), which is equivalent to the $\Gamma - K$ direction due to time-reversal. Red lines highlight the antisymmetric bands. Note that this direction has a C_2 rotation symmetry about y axis. The bands with rotation eigenvalue of -1 and 1 are plotted in black and red. Supplementary Figures 5b and 5c show the E_z field patterns of these bands.

Supplementary Note 4

Simulated surface transmissions

Here we calculate the surface transmission with a fixed k_z of $0.3\pi / d$, rather than a fixed incident angle as in our measurement. The simulation configurations are the same as that in Fig. 7. The surface transmissions are calculated by integrating the electric field strength at the right end of the surface, since our surface transmissions are measured by the receiving horn at the right end. Results are shown in Supplementary Fig. 8. The cyan box highlights the nontrivial bandgap predicted by the calculated band structure with $k_z = 0.3\pi / d$. Inside the nontrivial bandgap, the three curves almost overlap with each other indicating k_z-preserved one-way transport. Outside the bandgap, EM waves can propagate in the bulk of the crystal. The defect near the surface will affect the field pattern in the sample (also the field near the right end) but not necessarily block the signal because this is a complicated multiple reflection process when the waves propagate inside the crystal. Therefore the transmission with defect (green or violet curve) can be either larger or smaller than the one without defect (black curve) at different frequency. For instance, the green and violet curves are larger than the black curve above 13 GHz while they are smaller in the frequency region from 11.4 to 11.8 GHz. The simulated

transmissions in Supplementary Fig. 8 are calculated for a fixed k_z rather than a fixed incident angle in our experiment. Another reason for the discrepancy between the simulated spectra and the experimental results is that periodic boundaries are used in the z-direction in the simulations and the finite periods of the structure and the finite width of the incident beam are not taken into account.
